# Some Account of the African Remittent Fever Which Occurred on Board Her Majesty's Steam-Ship Wilberforce, in the River Niger, and Whilst Engaged on Service on the Western Coast of Africa; Comprising an Inquiry into the Causes of Disease in Tropical Climates

**Published:** 1843-10

**Authors:** 


### PART SECOND.

### Bibliographical Notices.

Art. I.—*Some Account of the African Remittent Fever which occurred on board Her Majesty's Steam-ship Wilberforce, in the River Niger, and whilst engaged on Service on the Western Coast of Africa; comprising an Inquiry into the Causes of Disease in Tropical Climates.* By Morris Pritchett, m.d. f.r.g.s., Member of the Royal College of Physicians of London, Fellow of the Royal Medical and Chirurgical Society, late Surgeon of H.M. Ship Wilberforce, &c.—*London*, 1843. 8vo, pp. 216.

This book reached us at too late a period, last quarter, to allow us to notice it at the same time with Dr. M'William's work on the same subject. Although badly arranged and exhibiting a good deal of loose speculation and hypothesis, Dr. Pritchett's volume is very creditable to himself and to the service to which he belongs. It contains full, and no doubt accurate details of everything relating to the fever as it occurred in the Wilberforce, and, together with Dr. M'William's book, must be consulted by all future explorers of the African rivers. The valuable matter contained in Dr. Pritchett's volume would be much more useful to the reader, if the author had taken the trouble to put it in more formal book-shape, by dividing it into chapters and sections, and giving us a table of contents. As it is, we have no guide to help us through its mazes.

Out of the 184 pages of text, of which Dr. Pritchett's volume consists, nearly 100 pages are given to a detail of the cases (43 in number,) which occurred under the author's care. These give a sufficiently good view of the disease, but present no feature of novelty. The disease was evidently the same as that described by Dr. M'William, the ordinary remittent of warm climates. From Dr. Pritchett's disregard of statistics, it is not always easy to come at results, but it would appear that the cases of fever occurring in the Wilberforce were sixty of remittent and twelve of intermittent, in all 72; and that of this number, it appears from the table in Dr. M'William's work, that eleven died. We find no novelties in the post-mortem appearances; and the following is the only novelty in the *treatment*, which we recommend to the consideration of our readers:

"The best means of lessening the sense of burning heat which was so commonly complained of, I found to consist in encouraging the natural process of perspiration by keeping the body somewhat warmly wrapped up, while the patient was inhaling the open air, as cool as it could be had; the sick in the Wilberforce were placed under the awning on the upper deck. So profuse was the cuticular discharge under these circumstances, that the bedding commonly soon became saturated with the excretion. This plan of treatment was adopted from observing that, when an attempt was made to keep the surface cool by constant sponging with cold water, or vinegar and water lotions, and removing the usual covering, the skin always became dry and parched, and the patient obviously uneasy and nervous. Those, on the contrary, who were treated in the way indicated above, lay quiet and bathed in perspiration, which evidently had a soothing effect on the whole system. On several occasions the warm bath was had recourse to; but any benefit resulting from it was by no means generally apparent." (pp. 167-8.)

Dr. Pritchett found bloodletting injurious; mercurialization at least useless; and quinine "not attended by that success which was anticipated." Some of our readers will smile at the following practical commentary on the author's statements, that "anything like activity, whether depletory or in the way of stimulation, was equally attended by an aggravation of the malady;" and that "the practice about to be recommended will probably be viewed by the advocates of either of these remedial plans, as *weak and wanting in energy*:"

"Where derangement of the chylopoetic organs is obviously present, the stomach ought to be emptied of its contents, and spasm of the hepatic ducts, if such exist, removed by the exhibition of an emetic; patients generally expressed themselves greatly relieved by its operation. A scruple of calomel, followed by a dose of castor oil, is next to be administered, with a view to clearing out the bowels. Should this not have proved efficacious, recourse may then be had to more active cathartics, such as croton oil, till the evacuations are sufficiently copious. These measures were followed up in the earlier period of the disease with doses of from five to ten grains of calomel, combined with three or four of the pulvis Jacobi veri, every two, three, or four hours, according to the urgency of the symptoms, till the mouth became slightly affected, when the medicine was discontinued." (pp. 165-6.)

A considerable portion of the work is occupied by an exposition of the author's views as to the causes and nature of the African fever. He strongly combats the common notion of the cause being *malaria*, or an unknown extraneous poison originating in particular localities; and endeavours to show that the cause is to be found in the ordinary atmospheric influences of hot climates. He seems to regard the *moisture* of a warm atmosphere, by inducing important changes in the system, as the great predisposing cause, and direct exposure to the solar rays as the great exciting cause of the African fever, both acting more or less through the medium of electrical changes produced in the system. We think, however, that Dr. Pritchett has altogether failed to make good any of these positions; and although the theory of the miasmatic origin of fever has received a severe blow from some facts recently disclosed by Dr. Wilson and Major Tulloch, this cannot be said to be strengthened by anything advanced in the present work.

